# Cross-cultural adaptation and psychometric validation of the Mindful Eating Scale in overweight and obese Chinese individuals

**DOI:** 10.3389/fpubh.2026.1825485

**Published:** 2026-06-04

**Authors:** Yucen Gu, Ziyu Sun, Yuhong Wu

**Affiliations:** 1 Zhejiang Cancer Hospital, Hangzhou Institute of Medicine (HIM), Chinese Academy of Sciences, Hangzhou, China; 2 School of Nursing, Hangzhou Normal University, Hangzhou, China

**Keywords:** Chinese, mindful eating, overweight and obesity, psychometric validation, scale

## Abstract

**Objective:**

This study aimed to adapt the Mindful Eating Scale (MES) for use in China and to assess its psychometric properties among individuals who are overweight or obese.

**Methods:**

The MES was translated and back-translated following the modified Brislin translation model. After expert review and pretesting, the scale underwent cross-cultural adaptation. Study participants were recruited from individuals who are overweight or obese and admitted to the endocrinology outpatient department of a hospital in Hangzhou. The psychometric properties of the cross-culturally adapted MES were subsequently evaluated.

**Results:**

A total of 221 questionnaires were distributed, of which 217 were returned, yielding a valid response rate of 98.19%. The cross-cultural adaptation of the MES consisted of 6 dimensions and 23 items. It demonstrated a Cronbach’s *α* coefficient of 0.905, an item-total correlation reliability of 0.833, and a retest reliability of 0.915. The chi-square-to-degrees-of freedom ratio (*χ^2^*/*df*) was 1.954, the root mean square error of approximation (RMSEA) was 0.066, the goodness-of-fit index (GFI) was 0.862, the adjusted goodness-of-fit index (AGFI) was 0.818, the comparative fit index (CFI) was 0.964, and the Tucker–Lewis index (TLI) was 0.956.

**Conclusion:**

The cross-culturally adapted MES demonstrated satisfactory psychometric properties. However, because dietary habits vary across regions and this study was limited to a specific region of China, further research is needed to determine whether the MES is a suitable instrument for assessing mindful eating levels among Chinese individuals who are overweight or obese.

## 1 Introduction

Overweight and obesity are global public health concerns (1). The “Report on the Nutritional Status and Chronic Disease Status of Chinese Residents (2020)” indicates that more than half of the Chinese adult population is overweight or obese, with unhealthy dietary behaviors considered the primary cause (2). Numerous studies have shown that most dietary intervention methods at home and abroad are led by healthcare professionals and focus only on improving patients’ unhealthy dietary behaviors, neglecting differences in patients’ psychological characteristics. Patients often experience psychological issues such as anxiety and depression (3). Therefore, it is essential to explore a sustainable self-management dietary intervention method that not only helps people improve their unhealthy dietary behaviors but also promotes mental health.

Mindfulness is a method of self-regulating attention, intended to direct one’s focus toward the present moment, enhance self-awareness, and enable individuals to respond to, analyze, and judge the current situation (4). Numerous studies have shown that mindfulness training can effectively reduce psychological stress, anger, neuroticism, cognitive repression, dissociation, depression, and anxiety (5). The application of mindfulness to regulate eating habits is known as mindful eating (6), which involves enhancing self-awareness through meditation during the eating process. Studies have shown that mindful eating can improve maladaptive eating behaviors such as picky eating, binge eating, and anorexia, especially those regarding eating behaviors driven by negative emotions (7). The Mindful Eating Scale (MES) is an effective multidimensional psychological measurement tool used abroad to measure the level of mindfulness in eating behaviors (8). Compared with other mindfulness-based eating assessment tools, the MES encompasses a more comprehensive range of mindfulness-related content. In studies involving overweight women and university students, only the MES showed significant changes following mindfulness-based eating interventions, with corresponding increases in scores (9, 10). Domestic research on the MES has been conducted (11, 12), but it has not yet been applied to individuals who are overweight or obese. The original MES is universally applicable. Its developer anticipated that this measure would be of use in the further development of mindfulness-based interventions for eating disorders and obesity. Thus, it’s important to select overweight and obese individuals as our research subjects.

Therefore, this study focuses on the Mindful Eating Scale (MES) in Chinese and explores the current status and influencing factors of mindful eating levels among individuals who are overweight or obese, with the aim of developing effective intervention measures to improve the quality of nursing services, reduce medical burdens, and improve patients’ dietary behaviors to promote their physical and mental health.

## 2 Methods

### 2.1 Study design

This study was carried out in two phases. First, a cross-culturally adapted MES was developed through initial translation and cross-cultural modification. Second, a cross-sectional survey was conducted to evaluate the psychometric properties of the cross-culturally adapted MES.

### 2.2 Ethical considerations

The Ethics Committee of Hangzhou Normal University approved the study (approval number: 2023050). Official permission was obtained through email from the researcher who developed the scale to adapt it into Chinese and use it in this study. The research adhered to the Helsinki Declaration of Human Rights. This study was conducted anonymously and on a voluntary basis for participants, and the data collected were used solely for academic purposes. Participants had the right to withdraw from the study at any time without incurring any additional risk or discomfort.

### 2.3 Cross-cultural adaptation of the MES

Under the authorization of the original author, we conducted forward-backward translation, cultural correction, and pretesting. The specific steps are as follows: (1) Direct translation: In accordance with the Brislin translation model (13), two researchers (producing MES-1 and MES-2) independently performed the translation. The two versions were compared, and the better version was selected to form the Chinese version of the MES-I. If neither version was acceptable, another translation was performed. (2) Back translation: A PhD holder and a university English teacher independently back-translated the first draft of the Chinese version of the MES into English (resulting in MES-3 and MES-4) without access to the original scales. (3) Integration: A master’s student compared and analyzed MES-3 and MES-4 with the original scale. They were adjusted and modified based on the differences between the two to form MES-II (Table 1). (4) Cross-cultural adaptation: A panel of five experts participated in two rounds of Delphi expert consultations (14) (Table 2). (5) Pretesting: Thirty participants were recruited to determine whether there were any semantically confusing and difficult-to-understand items on the scale. If more than 20% of participants found an item unclear, it was evaluated and revised. Modifications were made to some items based on participant feedback. Ultimately, the final Chinese version of the MES-III was finalized.

**Table 1:** Basic characteristics of the scale translator.

ID	Undertaken work	Educational background	Occupation	Bilingual experience
1	Direct translation	Master’s degree	Student	Master of English, TEM-8^a^
2	Direct translation	PhD	Professor	Overseas study experience
3	Back translation	Master’s degree	Professor	University English teacher
4	Back translation	PhD	Student	Master of nursing
5	Integration	Master’s degree	Student	Master of nursing, CET-6^b^

a Test for English majors grade eight.

b National college English test band 6.

**Table 2:** Basic information on the members of the panel.

ID	Educational background	Title	Professional background	Working years
1	PhD	Senior professional title	Psychology	13
2	PhD	Medium-grade professional title	Nutriology	23
3	PhD	Associate senior title	Nutriology	12
4	Master’s degree	Associate senior title	Clinical nursing	29
5	Master’s degree	Associate senior title	Endocrinology	15

### 2.4 Validation of the scale via a cross-sectional study

#### 2.4.1 Study setting and participants

Convenience sampling and snowball sampling were used to recruit individuals who are overweight or obese. To facilitate project analysis and exploratory factor analysis while maintaining stability, the sample size should be at least 5 to 10 times the number of items (15). The questionnaire used in this study consisted of 28 items. To account for potential data loss or invalid questionnaires during data collection, the sample size was expanded by 10% at a 1:5 to 1:10 ratio. Consequently, the study sample size ranged from 154 to 308 participants. The inclusion criteria were as follows: (1) a BMI ≥ 24.0 kg/m^2^ (16); (2) age ≥18 years; (3) clear consciousness and the absence of language or communication barriers; and (4) voluntary participation. The exclusion criteria were as follows: (1) previously or currently having impaired cognitive diseases such as mental or cognitive disorders, (2) taking glucocorticoids or other hormone medications, and (3) having a language communication disorder.

#### 2.4.2 Instruments

##### 2.4.2.1 Demographic data questionnaire

The demographic information included age, sex, marital status, educational background, financial situation, body mass index (BMI), disease status, and knowledge of mindfulness practices.

##### 2.4.2.2 The cross-culturally adapted MES

This scale consists of 6 dimensions with 28 items: acceptance, awareness, non-reactivity, acting with awareness, routine, and unstructured eating. The scale uses a 4-point Likert scale: never = 1, sometimes = 2, often = 3, and usually = 4. The average score of each dimension was calculated. A higher score reflected an individual’s higher level of mindfulness with regard to eating. In the original study, the Cronbach’s alpha coefficients for five of the six dimensions ranged from 0.71 to 0.89. The six dimensions of unstructured eating have an alpha of 0.60.

### 2.5 Data analysis

SPSS 26.0 and AMOS 24.0 were used for data analysis. The enumerated data are summarized using frequency and proportion. Descriptive statistics include the mean±standard deviation for normally distributed data and the median with the interquartile range for non-normally distributed data. The content validity, structural validity, and convergent validity were assessed. Content validity was estimated at the item level (I-CVI) and scale level (S-CVI). Five experts responsible for cross-cultural adjustment evaluated whether the items were aligned with the content being tested. Structural validity is evaluated via exploratory factor analysis (EFA) and confirmatory factor analysis (CFA). The average variance extracted (AVE) was used to assess discriminant validity. Reliability analysis used test–retest reliability, Cronbach’s alpha, and split-half reliability. Two weeks later, 20 participants were randomly selected for a follow-up survey to assess test–retest reliability. Differences with a *p*-value <0.05 were considered statistically significant.

## 3 Results

### 3.1 Cross-cultural adaptation of the MES

The experts assessed the Chinese translation of the original English version of the scale and provided recommendations for revising the items. The expert reliability coefficient (Cr) was 0.82. The expert review and the results after revision are shown in Table 3.

**Table 3:** Expert review and revision results.

Item	Original scale item	Revised scale item
Dimension 4	Routine	Dietary routine
Dimension 5	Act with awareness	Conscious eating behavior
Item 1	I criticize myself for the way I eat	I sometimes tend to blame myself for the choices I make regarding food
Item 4	I wish I could control my eating more easily	I wish I could have more ease in controlling my diet
Item 5	I tell myself I should not be hungry	I remind myself I should not feel hungry
Item 7	I notice flavors and textures when I’m eating my food	I notice taste and textures when I’m eating my food; set as Item 9
Item 8	I stay aware of my food while I’m eating	set as Item 7
Item 9	I notice how my food looks	set as Item 8
Item 13	Once I’ve decided to eat, I have to eat straight away	Once I want to eat, I feel the need to eat immediately
Item 15	I become very short-tempered if I need to eat	My temper becomes impatient when I need to eat
Item 16	I need to eat like clockwork	I need to eat meals on time
Item 17	I have a routine for what I eat	I have my own preferences regarding what to eat
Item 18	I have a routine for when I eat	I have established my own routines regarding when to eat
Item 21	I snack without being aware that I’m eating	I tend to unconsciously snack on snacks
Item 22	I do not pay attention to what I’m eating because I’m daydreaming, worrying, or distracted	Due to zoning out, worrying, or being absent-minded, I do not pay attention to what I am eating
Item 23	I eat automatically without being aware of what I’m eating	I eat without being aware of what I am consuming
Item 25	I multitask while eating	I eat while I’m doing something else

### 3.2 Pilot survey

The participants faced certain difficulties in comprehending “eating snacks,” “between meals,” and Item 23. The modifications were as follows: (1) change “between meals” to “between meals (e.g., between breakfast and lunch)”; (2) change Item 23 to “I eat something habitually”; (3) add a specific explanation for “snacking,” which refers to all foods except three meals or planned snacks, including fruits, dairy products, coffee, pasta, candy, nuts, beverages, and fried foods; and (4) change Item 18 to use positive scoring.

The final version of the scale, with 28 items and 6 dimensions, was formed, and the translator was asked to back-translate the revised Chinese version into English. The revised Chinese version and the back-translated English version of the scale were emailed to the author of the original scale to solicit their opinions. The final version of the cross-culturally adapted MES was formed after consent was obtained.

### 3.3 Characteristics of the participants

A total of 221 questionnaires were distributed in this study, resulting in 217 valid responses after excluding 4 invalid responses, yielding an effective response rate of 98.19%. The participants included 98 males (45.2%) and 119 females. The median age of the participants was 50 years. Educational attainment included high school/technical secondary school graduates (37.8%) and college/bachelor’s degree holders (41.9%), with the predominant income range of 3,000–5,000 yuan reported by 46.5% of participants.

All participants’ BMIs were greater than 24 kg/m^2^, with a median BMI of 28 kg/m^2^. Notably, approximately 42% of participants reported having no other medical conditions; however, the remainder presented with chronic diseases such as hypertension, diabetes, and fatty liver disease. With the exception of a few participants, most did not engage in mindfulness practices (95%).

### 3.4 Item analysis

In this study, item-total correlations, reliability changes, and discrimination values were used to filter the items. The exclusion criteria included an item–total correlation below 0.3, an increase in reliability after item deletion, and a discrimination value below 3 between high and low groups. Items 3 (‘I am accustomed to assessing whether my diet is reasonable’), Item 6 (‘I wish I could control my hunger’), and Item 16 (‘I need to eat at regular times’) did not significantly differ, with discrimination values of 1.906 (*p* > 0.05), 1.422 (*p* > 0.05), and 0.256 (*p* > 0.05), respectively, between the high and low groups. After deletion, the reliability of the scale increased to 0.893 and 0.894, respectively. Items 3, 6, and 16 were eliminated, and ultimately, 25 items were retained. The specific analysis results are shown in Table 4.

**Table 4:** Item analysis of the cross-culturally adapted MES.

Item	Discrimination value	*p*-value	Item-total correlation	α after item deletion	Conclusion
1	11.506	<0.001	0.644	0.88	Reserve
2	12.676	<0.001	0.696	0.879	Reserve
3	1.906	0.059	−0.046	0.893	Delete
4	10.778	<0.001	0.655	0.88	Reserve
5	16.689	<0.001	0.760	0.876	Reserve
6	1.422	0.158	−0.063	0.893	Delete
7	11.144	<0.001	0.662	0.88	Reserve
8	11.701	<0.001	0.629	0.881	Reserve
9	9.747	<0.001	0.568	0.883	Reserve
10	9.195	<0.001	0.584	0.882	Reserve
11	11.352	<0.001	0.638	0.881	Reserve
12	5.772	<0.001	0.367	0.887	Reserve
13	6.7	<0.001	0.460	0.886	Reserve
14	7.412	<0.001	0.500	0.884	Reserve
15	7.176	<0.001	0.485	0.885	Reserve
16	0.256	0.798	−0.04	0.894	Delete
17	6.456	<0.001	0.423	0.887	Reserve
18	4.911	<0.001	0.357	0.888	Reserve
19	3.726	<0.001	0.335	0.887	Reserve
20	3.236	0.002	0.329	0.887	Reserve
21	6.9	<0.001	0.408	0.886	Reserve
22	7.012	<0.001	0.397	0.886	Reserve
23	7.416	<0.001	0.412	0.886	Reserve
24	7.002	<0.001	0.398	0.886	Reserve
25	16.291	<0.001	0.744	0.877	Reserve
26	11.646	<0.001	0.693	0.88	Reserve
27	14.465	<0.001	0.731	0.878	Reserve
28	17.154	<0.001	0.793	0.875	Reserve

### 3.5 Validity test

#### 3.5.1 Content validation

Using a 4-point Likert scale, five experts in relevant fields evaluated the I-CVI and the S-CVI. The results indicated that, except for Items 19 and 20, all remaining I-CVIs exceeded 0.8, while the S-CVI was calculated at 0.89. Specifically, Item 19 (“I eat the same food on the same day every week”) and Item 20 (“I eat the same food for lunch every day”) received scores of 1 from two experts and were subsequently removed. Following this deletion, the I-CVI increased to 1.00, and the S-CVI rose to 0.93—both values exceeded the standard threshold of 0.9.

#### 3.5.2 Structural validation

##### 3.5.2.1 Exploratory factor analysis (EFA)

Bartlett’s sphericity test yielded a result of 5564.910 (*p* < 0.001), indicating a significant correlation among the variables. The Kaiser–Meyer–Olkin (KMO) test value for the exploratory factor analysis was 0.859, indicating that the data were suitable for factor analysis. Principal component analysis was used to extract common factors, retaining those with eigenvalues ≥1.00. The analysis revealed the extraction of 6 common factors, consistent with the original scale. The explained variance of the six dimensions was as follows: 35.831% for dimension 2, 17.962% for dimension 5, 12.866% for dimension 3, 9.89% for dimension 6, 5.56% for dimension 1, and 4.657% for dimension 4. The cumulative explained variance was 86.768%. The factor loading results for the 23 items are presented in Table 5.

**Table 5:** KMO of the cross-culturally adapted MES.

Item	Dimension 1	Dimension 2	Dimension 3	Dimension 4	Dimension 5	Dimension 6
Item 1	0.854					
Item 2	0.788					
Item 4	0.836					
Item 5	0.767					
Item 7		0.846				
Item 8		0.822				
Item 9		0.893				
Item 10		0.902				
Item 11		0.916				
Item 12			0.816			
Item 13			0.946			
Item 14			0.911			
Item 15			0.941			
Item 17				0.914		
Item 18				0.923		
Item 21					0.935	
Item 22					0.934	
Item 23					0.941	
Item 24					0.935	
Item 25						0.814
Item 26						0.857
Item 27						0.817
Item 28						0.819

##### 3.5.2.2 Confirmatory factor analysis

Using confirmatory factor analysis (CFA) to validate the fit of the scale structure, the results showed that the six-factor model had a good fit, with all indicators meeting the standard. The χ^2^/df value is 1.954, the GFI value is 0.862, the AGFI value is 0.818, the CFI value is 0.964, the TLI value is 0.956, and the RMSEA value is 0.066. The loadings of each item on its corresponding factor are greater than 0.7.

Furthermore, correlation analysis was conducted on each item of the scale, its corresponding dimensions, and the other dimensions. The discriminant validity of each factor was tested. The results revealed that the correlation coefficients between the items of the cross-culturally adapted MES and their respective dimensions were greater than those between the items and other dimensions (Table 6).

**Table 6:** Discriminative validity of each dimension of the cross-culturally adapted MES.

Dimension	Acceptance	Awareness	Non-reactivity	Routine	Act with awareness	Unstructured eating
Acceptance	0.795					
Awareness	0.558^**^	0.806				
Non-reactivity	0.219^**^	0.255^**^	0.800			
Routine	0.174^**^	−0.012^**^	0.165^**^	0.853		
Act with awareness	0.093^**^	0.066^**^	−0.024^**^	0.303^**^	0.851	
Unstructured eating	0.174^**^	0.444^**^	0.221^**^	0.338^**^	0.286^**^	0.789
AVE square root	0.892	0.898	0.894	0.924	0.922	0.888

^**^p < 0.01; the diagonal line represents the amount of variance extracted from the AVE evaluation.

### 3.6 Reliability analysis

The internal consistency reliability of the total score and each dimension of the cross-culturally adapted MES ranged from 0.905 to 0.964, the split-half reliability ranged from 0.883 to 0.977, and the test–retest reliability ranged from 0.844 to 0.958, indicating that the scale has good reliability. The internal consistency reliability, split-half reliability, and test–retest reliability of the total score and each dimension of the cross-culturally adapted MES are detailed in Table 7.

**Table 7:** Reliability test of the cross-culturally adapted MES.

Dimension	Number of items	Internal consistency reliability	Split-half reliability	Test–retest reliability
Acceptance	4	0.922	0.964	0.880
Awareness	5	0.952	0.952	0.958
Non-reactivity	4	0.937	0.945	0.937
routine	2	0.907	0.914	0.948
act with awareness	4	0.964	0.977	0.902
unstructured eating	4	0.928	0.936	0.844
Total score	23	0.905	0.833	0.915

## 4 Discussion

### 4.1 Discriminative validity of the cross-culturally adapted MES by item

This study encompasses the translation and revision of the MES into Chinese, resulting in a 23-item version across 6 dimensions. The outcomes of the item analysis indicate that Items 3, 6, and 16 have non-significant discriminative validity (*p* > 0.05), suggesting that these items do not effectively differ between respondents. In contrast, the discriminative values for the remaining items exceed 3.0, with statistically significant differences (*p* < 0.01). Furthermore, the correlation coefficients between the 23 items and the total score ranged from 0.329 to 0.793 (*p* < 0.01). Excluding Items 3, 6, and 16, the correlation coefficients for the other items were greater than 0.3, indicating good correlation. These results may be influenced by cultural differences between Eastern and Western contexts, resulting in difficulties for respondents in comprehending the content of these three items.

### 4.2 Reliability of the cross-culturally adapted MES

The cross-culturally adapted MES exhibits excellent reliability, with a Cronbach’s *α* coefficient of 0.905. However, research suggests that a Cronbach’s α coefficient of >0.90 may indicate item redundancy (17). The Cronbach’s α coefficients for each dimension are all above 0.7. The test–retest reliability is 0.915, indicating the scale’s strong measurement stability. This finding indicates that the translation process did not encounter significant issues and that each item effectively reflects the issues under investigation. Therefore, it can be concluded that the cross-culturally adapted MES is reliable and can serve as an effective instrument for evaluating mindful eating among individuals who are overweight or obese in Hangzhou, China.

### 4.3 Validity of the cross-culturally adapted MES

In this study, five experts from diverse fields were invited to assess the content validity of the scale. The findings showed that two experts considered Items 19 and 20 to be inconsistent with the dietary habits of Chinese individuals. After these items were eliminated, the content validity index (I-CVI) of the cross-culturally adapted MES was 1.00, and the scale-level content validity index (S-CVI) of the scale was 0.93, indicating good content validity. Exploratory and confirmatory factor analyses revealed that the structure of the 6 common factors met the fit index criteria, presenting satisfactory basic fit indices consistent with the principles of model identification. Additionally, there was a correlation between the common factors and the total score, reflecting excellent discriminative ability and representativeness.

The findings of this study imply that the cross-culturally adapted MES demonstrates good validity and can precisely assess the level of mindful eating among individuals who are overweight or obese in Hangzhou, China. These results are in line with those from studies carried out by the original authors of the scale on university students and Brazilian scholars investigating overweight women (8, 18). However, our results differ from those reported in studies conducted in Poland (19), Greece (20), and Japan (21), which might be attributed to differences in dietary habits and cultural backgrounds among various countries.

### 4.4 Clinical significance of the cross-culturally adapted MES

Mindfulness training not only effectively alleviates psychological stress and negative emotions, including anger, neuroticism, cognitive suppression, dissociation, depression, and anxiety (5) but also enables individuals to experience increased joy and satisfaction. Furthermore, it enhances mental health and resilience while improving emotional regulation, thereby helping individuals achieve their personal goals (22). This study provides a scientific foundation for guiding individuals who are overweight or obese toward healthier eating habits from a psychological perspective. It contributes to establishing positive dietary practices among this population, thereby reducing the incidence of chronic diseases associated with poor dietary habits.

## 5 Conclusion

The cross-culturally adapted MES demonstrates good reliability and validity, fulfilling the measurement requirements necessary for evaluating mindful eating levels among individuals who are overweight or obese in Hangzhou, China. However, this study has certain limitations: the sample was restricted to a single hospital in Hangzhou, a convenience sampling method was used, the BMI categories in other countries are not the same as those used in China (23), and dietary habits vary across different regions, which may limit the representativeness of the sample. Additionally, this study did not assess its calibration validity in the Chinese context. Confirmatory factor analysis is more appropriate for large sample statistical evaluations. Constraints related to time, financial resources, manpower, and material resources resulted in a sample size that met only the lower threshold required for structural equation modeling; this limitation may impact model fit quality. Future research should consider increasing sample sizes in clinical settings to further validate the psychometric properties of this scale and to explore additional applications.

## Data Availability

The raw data supporting the conclusions of this article will be made available by the authors, without undue reservation.
